# Longitudinal-Survival Models for Case-Based Tuberculosis Progression

**DOI:** 10.3389/fpubh.2021.543750

**Published:** 2021-04-19

**Authors:** Richard Kiplimo, Mathew Kosgei, Ann Mwangi, Elizabeth Onyango, Morris Ogero, Joseph Koske

**Affiliations:** ^1^School of Sciences and Aerospace Studies, Moi University, Eldoret, Kenya; ^2^National TB, Leprosy and Lung Disease Program, Ministry of Health, Nairobi, Kenya; ^3^KEMRI Wellcome Trust, Nairobi, Kenya

**Keywords:** Markov Chain Monte Carlo method, B splines, joint model, current value, tuberculosis

## Abstract

**Introduction:** Tuberculosis (TB) disease continues to be responsible for a high global burden with an estimated 10 million people falling ill each year and an estimated 1.45 million deaths. Widely carried out analyses to utilize routine data coming from this disease, and well-established in literature, have paid attention to time-to-event with sputum smear results being considered only at baseline or even ignored. Also, logistic regression models have been used to demonstrate importance of sputum smear results in patient outcomes. A feature presented by this disease, however, is that each individual patient is usually followed over a period of time with sputum smear results being documented at different points of the treatment curve. This provides both repeated measures and survival times, which may require a joint modeling approach. This study aimed to investigate the association between sputum smear results and the risk of experiencing unfavorable outcome among TB patients and dynamically predict survival probabilities.

**Method:** A joint model for longitudinal and time-to-event data was used to analyze longitudinally measured smear test results with time to experiencing unfavorable outcome for TB patients. A generalized linear mixed-effects model was specified for the longitudinal submodel and cox proportional hazards model for the time-to-event submodel with baseline hazard approximated using penalized B-splines. The two submodels were then assumed to be related via the current value association structure. Bayesian approach was used to approximate parameter estimates using Markov Chain Monte Carlo (MCMC) algorithm. The obtained joint model was used to predict the subject's future risk of survival based on sputum smear results trajectories. Data were sourced from routinely collected TB data stored at National TB Program database.

**Results:** The average baseline age was 35 (SD: 15). Female TB patients constituted 36.42%. Patients with previous history of TB treatment constituted 6.38% (event: 15.25%; no event: 5.29%). TB/HIV co-infection was at 31.23% (event: 47.87%; no event: 29.20%). The association parameter 1.03 (CI[1.03,1.04]) was found to be positive and significantly different from zero, interpreted as follows: The estimate of the association parameter α = 1.033 denoted the log hazard ratio for a unit increase in the log odds of having smear positive results. HIV status (negative) 0.47 (CI [0.46,49]) and history of TB treatment (previously treated) (2.52 CI [2.41,2.63]), sex (female) (0.82 CI [0.78,0.84]), and body mass index (BMI) categories (severe malnutrition being reference) were shown to be statistically significant.

**Conclusion:** Sputum smear result is important in estimating the risk to unfavorable outcome among TB patients. Men, previously treated, TB/HIV co-infected and severely malnourished TB patients are at higher risk of unfavorable outcomes.

## 1. Introduction

Tuberculosis (TB) continues to be responsible for a high global disease burden. According to (1), an estimated 10 million people fall ill each year while 1.45 million die. The fight against this disease has evolved through various strategies, which include directly observed treatment, short-course (DOTS), stop TB strategies, and now in the stretch of end TB strategy. The set targets are reduction in TB incidence rate by 90%, reduction in number of TB deaths by 95%, and zero catastrophic costs borne by TB-affected families (2). However, patients still experience unfavorable treatment outcomes (death and lost to follow up) partly as a result of long (6 months) treatment duration. These call for granular analyses of available data to explain the unfavorable treatment outcomes.

Since introduction of DOTS strategy in 1993, WHO recommended a standard set of data variables, which span demographics, clinical, bacteriological, and treatment outcome to be routinely collected. Additionally, non-routine data are collected through surveys. Submission of the routinely collected data to national TB programs (NTP) is usually in form of aggregate numbers or case based. The latter provides a unique opportunity for researchers seeking to guide policy formulation, which ensures better patient outcome through monitoring patient treatment curve (3).

The routine data, most of the time is collected during first visit, more often referred to as baseline, during repeat visits and also documentation of treatment outcomes of the administered treatment. These provide longitudinal together with time-to-event data.

Researches done in different countries, Kenya included, utilizing these TB data have explored the survival arm of the data using Cox model. Examples include (6–12), all which did not include sputum smear results in their model. Studies by (8, 13) considered only baseline sputum smear measures while (14) derived a variable on whether a patient had follow up sputum or not. Others studies (15, 16) used mixed-effect models to demonstrate importance of sputum results in patient outcomes.

(4), however, explains that research questions with such follow-up and time-to-event outcomes could require separate analysis, most of the time interest being on joint analysis. To understand this, there is need for clear distinction between endogenous and exogenous covariates.

In TB setting, sputum smear results of the same patient are stochastically generated through the treatment course and these are most likely related to the event. That is, the existence of sputum result and/or its future path is informative about the occurrence (or non-occurrence) of an event (27). This means that inclusion of the whole longitudinal history might be key in providing an understanding on evolution of the disease over time. Separate analysis of such data therefore maybe potentially insufficient as they do not put into consideration endogeneity (4, 5).

Joint modeling, which in statistical literature refers to methods for simultaneously analyzing longitudinal and time-to-event models has witnessed an explosion in the recent years (4, 17–19). The benefits of postulating such models being explicit acknowledgment of endogeinity and measurement errors in repeated measures and also obtaining accurate estimate of the association between longitudinal and event processes (27). These have been applied in the field of epidemiology to model complex data with features such as multiple longitudinal outcomes (20), recurrent events (21) and longitudinal and time to event data (22). The settings for application are discussed by (19, 20, 22, 23). These include; when interest is on time-to-event outcome and we wish to include longitudinally measured variable, when the focus is on the longitudinal outcome and we wish to correct for nonrandom informative dropout, when interest is in the association between longitudinal and survival processes and lastly, when focus is on using longitudinal history to dynamically predict survival probabilities.

Examples of research that have adopted joint modeling methods and extensively published include (4, 24–26). Important ingredients are the different association structures (the current value parameterization, random effect parameterization, and lagged parameterization among others) discussed in (4). Under the current value parameterization (which was utilized in this study), the risk for an event at a particular time point *t* depends on the true level of the longitudinal marker at the same time point. Also discussed are parameter estimation techniques that include frequentist approach and Bayesian approach. This research therefore proposed fitting joint modeling in a specific application in TB to demonstrate how the sputum smear results of the TB patient recorded at fixed time points within the observation period together with baseline covariates recorded at the start of treatment can be effectively used to answer our important epidemiological questions. **JMbayes** package in R was chosen for these analyses because of its flexibility to model the association structure and its capability to fit joint model for categorical repeated measures (27, 28).

The objectives of the study were to investigate the association between sputum smear and the risk of unfavorable outcome and to predict individual's survival probability.

To the best of our knowledge, this is the first study to:

- Jointly model the association between sputum smear result, which is a repeated measure and the risk of unfavorable outcome using mixed effects logistic regression model and cox model with specified hazard embedded;
- Establish the possibility of predicting individual's survival probability during treatment, hence providing better understanding of TB patients' risk of unfavorable outcome.

This paper is structured as follows. In section 2, we provide the study design, source of data, and joint model formulation. Section 3 describes the data and the joint model output and dynamic predictions. We give the discussions and conclusion in section 4.

## 2. Materials and Methods

### 2.1. Study Design, Area, and Population

This was a retrospective cohort study. The target population was all people who had been notified as having tuberculosis disease in the 3 years (2014, 2015, and 2016) of our study to the NTP of Kenya.

### 2.2. Tuberculosis Data

Data on TB cases reported in the years 2014, 2015, and 2016 were used in this study. These were sourced from TIBU (Tuberculosis Information Basic Unit), the national electronic web based system at the NTP. Details on TIBU are given elsewhere (29, 30) but in brief, TIBU, a case-based electronic data collection system is a digital solution dedicated to digitalizing sustainable lung health reporting and routine surveillance in Kenya (29). It is an android-based application running on hand-held devices and stores data online, which is accessible via the internet. Currently, TIBU is being used by over 350 county and sub-county coordinators country. The system has inbuilt validation checks and provide real-time TB data at all levels of the health system.

At the facility level, once a patient is diagnosed and initiated on treatment, his/her details that include age, sex, type of TB, treatment outcome, date of start of treatment, HIV status, sputum smear results, treatment outcome, and date of treatment outcome among other variables are documented in the facility register. Sub-County TB and Leprosy Coordinators (SCTLCs) then transcribe these patient details from the TB facility register to tablet computers that run on android operating system. The case-based data are then transmitted directly to the national database (TIBU) via the mobile network.

These data were exported from the TIBU system into analyzable format in an excel sheet. This was then imported into R software for data cleaning and analyses. The analyses were based on all TB cases meeting the inclusion criteria after data cleaning process.

The variables of interest were unfavorable outcome (derived from the treatment outcome variable by combining patients who were lost to follow up or had died) and sputum smear results (a repeated measure).

Patients who were diagnosed and had initial sputum smear results of either “positive” or “negative” were included in the study. Patients who were diagnosed as extra pulmonary (EPTB) or had their initial smear as not done were excluded from the study. Also patients who did not have their HIV status documented or body mass index not documented were excluded.

#### 2.2.1. Operational Definitions

TB treatment outcome is defined as the final outcome a TB patient is assigned at the course of treatment or at the end of treatment. Definitions of various treatment outcomes that include cured, treatment complete, failure, loss to follow up, and died are explained in (31). In this study, they were categorized as favorable and unfavorable treatment outcomes. Favorable outcomes included cured and treatment complete cases. Unfavorable outcomes included people who had been assigned outcomes of loss to follow up and died.

Sputum smear results are the test results for the sample of sputum collected from persons presumed to have TB. This is done at the point of diagnosis usually denoted as month 0, at month 2, at month 5, and at month 6 of TB treatment. The results are usually classified as “positive” or “negative.” Patients not able to expectorate can be initiated on treatment based on clinical symptoms and have their sputum results documented as “not done.” Two outcome variables are of interest: The event time which is the time until an event is experienced, i.e., time until a TB patient initiated on TB treatment experiences unfavorable outcome. Second is the smear results emanating from sputum smear sample collected repeatedly from TB patient at diagnosis and during treatment follow up.

Smear conversion refers to change of smear results from positive in month 0 to negative in the second month of treatment.

### 2.3. Non-informative Assumption

A key assumption described by (4) is that censoring mechanism and the visiting process are independent of the true event times and future longitudinal measurements. The visiting process has been defined in literature as mechanism that provides time points where repeated measures are taken (4) usually classified as fixed or random, whereas the study's process is fixed at 0, 2, 5, and 6 months, little variability in the visiting times existed. Under the non-informative assumption, the visiting process was ignored without influencing the asymptotic properties of parameter estimations. This implied missing at random (MAR) assumption.

### 2.4. Joint Modeling

Joint modeling for longitudinal and time-to-event data is the joint estimation of a longitudinal mixed effect model, which analyzes patterns of change in an outcome variable that has been measured repeatedly over time, and a survival model, which analyzes the time until an event of interest occurs. Joint estimation is achieved by assuming the submodels are correlated through individual-level random effects, i.e., the longitudinal and the survival submodels share the same random effects (4). Both classical and Bayesian approaches to joint modeling have been considered with latter being especially useful because of its flexibility in fitting a wide range of joint models including joint models for categorical repeated measures (27) hence the choice of approach in this research.

#### 2.4.1. Model Formulation

#### 2.4.2. Generalized Linear Mixed Modeling

Let *y*_*ij*_ correspond to the observed value of the biomarker for individual *i*(*i* = 1, …, *N*) and each individual has *j* = 1, …, *n*_*i*_ repeated observations. The outcome variable *y*_*ij*_ = 1 for a positive response and *y*_*ij*_ = 0 for a negative response.

Modeling of binary outcome variable assumes that *y*_*ij*_ follows a distribution in the exponential family. The presence of repeated measures introduces random effects in the model. The random effects are incorporated within patient correlation and between patient variation, resulting in generalized linear mixed model (GLMM) (32) we therefore considered a GLMM.

That is,

$$\begin{array}{c} \;y_{ij}=g(\mu_{ij})\\ g[E(Y_{i}j|X_{i}j,b_{i})])=X_{ij}^{T}\beta +Z_{ij}^{T}b_{i}, \end{array}$$

where

*g*(.) is a known link function

*μ*_*ij*_ is the mean of the exponential family,

*y*_*i*_ is *n* × 1 vector of observed response values,

*β* is the *p* × 1 vector of fixed-effects parameters and have subject specific interpretations,

*X*_*i*_ is the *n*_*i*_ × *p* observed design matrix corresponding to the fixed effects,

*b*_*i*_ is the *q* × 1 vector of random-effects parameters,

*Z*_*i*_ is the *n*_*i*_ × *q* observed design matrix corresponding to the random effects.

#### 2.4.3. Assumptions

1.
  $$b_{i}\sim Normal\;(0,D),$$

where *D* is the covariance matrix for *b*_*i*_.

The expression for *E*(*Y*_*i*_*j*|*X*_*i*_*j*), does not, in general, have a closed-form expression. This basically means that in principle maximum likelihood is applied when making inferences, the application here is difficult due to intractable integrals. This led to the use of the penalized quasi-likelihood method (33) to estimate the parameters and predict the random effects.

#### 2.4.4. Event Submodel

Survival analysis is a method for analyzing data where outcome variable of interest is time to experiencing an event. In practice, the key guide for consideration of survival analysis is that the survival times have skewed distribution and that some subjects may never experience the event, referred to as censoring. Survival analysis allows for modeling of the effect of baseline covariate on the hazard of an event occurring. Specifically, the cox model is used to explain the risk that an individual will experience an event at a given time. It relies on proportionality assumption of the hazards. The present work assumes that censoring is non-informative. Also, the type of censoring is right censoring as some patients had not experienced the event of interest by end of treatment.

Henceforth, $T_{i}=\text{min }\left(T_{i}^{*},C_{i}\right)$ denotes observed event time where $T_{i}^{*}$ is the “true” event time for individual *i* and *C*_*i*_ denotes the censoring time.

$d_{i}=I\left(T_{i}^{*}\leq C_{i}\right)$ denotes the event indicator

The Cox proportional hazard model as proposed by (34) expresses the hazard of an event at time *t* as:

$$\begin{array}{l} h_{i}\left(t\right)=h_{0}\left(t\right)exp\left(\omega^{T}\gamma \right), \end{array}$$

where *ω* is the matrix of baseline covariates, which are assumed to be associated with the hazard of each subject. These included age, sex, HIV status, body mass index (BMI) category, and treatment history;

*γ* is the vector of fixed effect parameters;

*h*_0_(*t*) is the baseline hazard or baseline risk function where the effects of covariates are zero.

From our model, the baseline risk function as per the cox model is unspecified that might lead to an underestimation of the standard errors of the parameter estimates (4, 35). Several options that correspond risk function to a known parametric distribution have been defined in literature. These included weibull, log-normal, gamma, piecewise-constant, and regression splines (4). B-splines, which are piecewise polynomials whose pieces fit together smoothly along disease progression, was preferred because it provides a simpler, numerically more stable approach to approximating large amounts of data.

#### 2.4.5. The Joint Modeling Structure

Joint model describe the evolution of the biomarker in time for each patient. The estimated evolutions are then used in a survival model. The main aim of this study was to relate longitudinally measured smear test results with time to experiencing unfavorable outcome for TB-infected patient so as to understand the association between the two processes. The current value parameterization was used to capture the strength of the association between the current value of the longitudinally measured sputum smear results and the risk (4).

Let *m*_*i*_(*t*) be the true and unobserved value of the marker at time *t*. Then, a standard relative risk model as shown by (4) is as follows:

$$\begin{array}{l} h_{i}\left(t|M_{i}\left(t\right),\omega_{i}\right)=h_{0}\left(t;\omega \right)\text{exp }\left(\gamma^{T}\omega_{i}+\alpha m_{i}\left(t\right),\right), \end{array}$$

where *h*_*i*_(*t*) is the hazard of the event for individual *i* at time *t*; *m*_*i*_(*s*), 0 < *s* < *t* is the history of the true unobserved longitudinal process up to *t*;

*h*_0_(*t*; ***ω***) is a vector of baseline covariates with corresponding regression coefficients ***γ***. Here, unlike the cox model, the baseline risk function was approximated using penalized splines;

*α* is the strength of association between the biomarkers and the risk of an event (hazard).

#### 2.4.6. Conditional Independence Assumptions of Joint Model

Let *θ* denote the combined vector of all population-level parameters in the models

1. $$y_{i}\left(t\right)⊥y_{i}\left(t^{\prime }\right)|b_{i},\theta$$
  repeated measurements of the biomarker taken on the *ith* individual at two different times *t* and *t*′ are independent of one another.
2. $$y_{i}\left(t\right)⊥T_{i}^{*}|b_{i},\theta$$
  Any biomarker measurement for individual *i* is independent of that individual's true event time $T_{i}^{*}$
3. $$C_{i}⊥T_{i}^{*}|\theta$$
  that the censoring process for the event outcome is independent of the true event time.
4. Given the random effects, both the longitudinal and event time process are assumed independent, and the longitudinal responses of each subject are assumed independent.

### 2.5. Estimation of Joint Model Parameters

Inference of the parameters were based on posterior distribution. These were obtained under Bayesian theorem using Markov chain Monte Carlo (MCMC) algorithm.

Formally as described by (27)

$$\begin{array}{c} \;f(y_{i},T_{i},\delta_{i}|(b_{i},\theta )=p(y_{i}|b_{i},\theta )p(T_{i}|b_{i},\theta )\\ f(y_{i}|(b_{i},\theta )=\prod_{l}p(y_{il}|b_{i},\theta ) \end{array}$$

The posterior can be specified as

$$p(\theta ,b)\propto \left[\left(\prod_{i=1}^{n}\prod_{l=1}^{n_{i}}p(y_{ij}||b_{i},\theta )\right)p(T_{i},d_{i}||b_{i},\theta )p(b_{i}|\theta )p(\theta )\right]$$

which is equivalent to

$$\begin{array}{l} \text{log }p(\theta ,b_{i}|y_{i},T_{i},d_{i})\propto \left(\sum_{i=1}^{N}\sum_{j=1}^{n_{i}}\text{log }p(y_{ij}(t)|b_{i},\theta )\right)\\ +\text{log }p(T_{i},d_{i}|b_{i},\theta )+\text{log }p(b_{i}|\theta )+\text{log }p(\theta ), \end{array}$$

where,

$$\overset{n_{i}}{\underset{j=1}{\sum }}\log p\left(y_{ij}|b_{i},\theta \right)$$

is the log likelihood for the *mth* biomarker in the longitudinal submodel

$$\log p\left(T_{i},d_{i}|b_{i},\theta \right)=d_{i}*\log h_{i}\left(T_{i}\right)-\int_{0}^{T_{i}}h_{i}\left(s\right)ds$$

is the log likelihood for the event submodel,

where,

$$\int_{0}^{T_{i}}h_{i}\left(s\right)ds$$

is the cumulative hazard for individual *i*,

$$\log p\left(b_{i}|\theta \right)$$

is the log likelihood for the distribution of the individual-specific parameters (random effect), and log *p*(*θ*) is the log likelihood for the joint prior distribution of the unknown parameters.

We fitted the longitudinal submodel with the glmmPQL() function from the MASS package where the covariates (age, sex, HIV status, BMI category, and treatment history) were included. The random components were the individual patients who were followed through the treatment. We then, separately, fitted survival submodel with similar covariates. Finally, we combined both submodels considering the current value association structure between the two submodels.

As the integrals do not have a closed form solution, these were approximated numerically using MCMC algorithm provided in the R package called *JMbayes* (27). The plot function was used to examine convergence using JointModelBayes() MCMC estimation.

### 2.6. Predicted Survival in Joint Models

The obtained joint model was then used to derive survival predictions based on a set of longitudinal measurements $Y_{i}(t)=y_{i}(s);0\leq s\leq t$ for four patients sampled from study data and excluded before fitting the joint model (4, 19).

The focus on the conditional survival probabilities was therefore on time *u* > *t* given survival up to time *t*, i.e.,

$$\pi_{i}\left(u|\text{t}\right)=P\left(T_{i}^{*}>u|T_{i}^{*}>t,x_{i}\left(t\right),w_{i},D_{n};\theta \right)$$

where θ^*^ denotes the true parameter values, *D*_*n*_ is the fitted joint model, and *ω*_*i*_ is a vector of baseline covariates. Here, *π*_*i*_(*u*|*t*) is dynamic as and when new information about patient was obtained at time *t*′ > *t* (4). These were computed using the function survfitJM() available in JMbayes package in R. All the analyses were conducted using R programming language (36).

## 3. Results

A total of 131,809 cases were included in the study, of which 14,365 (10.90%) had experienced the event. Descriptive analyses used include mean, standard deviation, and proportion depending on the nature of baseline variable. As shown in Table 1, the mean age was 35 (SD: 15). Out of all the cases enrolled, female constituted 36.42% (event: 32.43%; no event: 36.91%). Patients with previous history of treatment constituted 6.38% (event: 15.25%; no event: 5.29%). TB/HIV co-infection rate was at 31.23% (event: 47.87%; no event: 29.20%). Under BMI classification, severely malnourished (SM) constituted 16.73%, moderately malnourished (MM) were 33.18%, 40.62% had normal BMI, 4.31% were overweight while obese patients constituted 5.15%.

**Table 1:** Descriptive statistics.

**Characteristic**	**Category**	**Overall**	**Event**	**No event**
		**(*****n*** **= 131,809)**	**(*****n*** **= 14,365)**	**(*****n*** **= 117,444)**
		**mean(SD)**	**mean(SD)**	**mean(SD)**
**Age**		**35 (15)**	**38 (15)**	**35 (14)**
		%	%	%
Sex	Male	63.58	67.57	63.09
	Female	36.42	32.43	36.91
HIV status	Pos	31.23	47.87	29.20
	Neg	68.77	52.13	70.80
Treatment history	New	93.62	84.75	94.71
	Previously treated	6.38	15.25	5.29
BMI category	Severely mal	16.73	25.13	15.71
	Moderately mal	33.18	34.92	32.97
	Normal	40.62	34.72	41.34
	Overweight	4.31	3.01	4.47
	Obese	5.15	2.23	5.51

### 3.1. Joint Model

**Figure 1:**
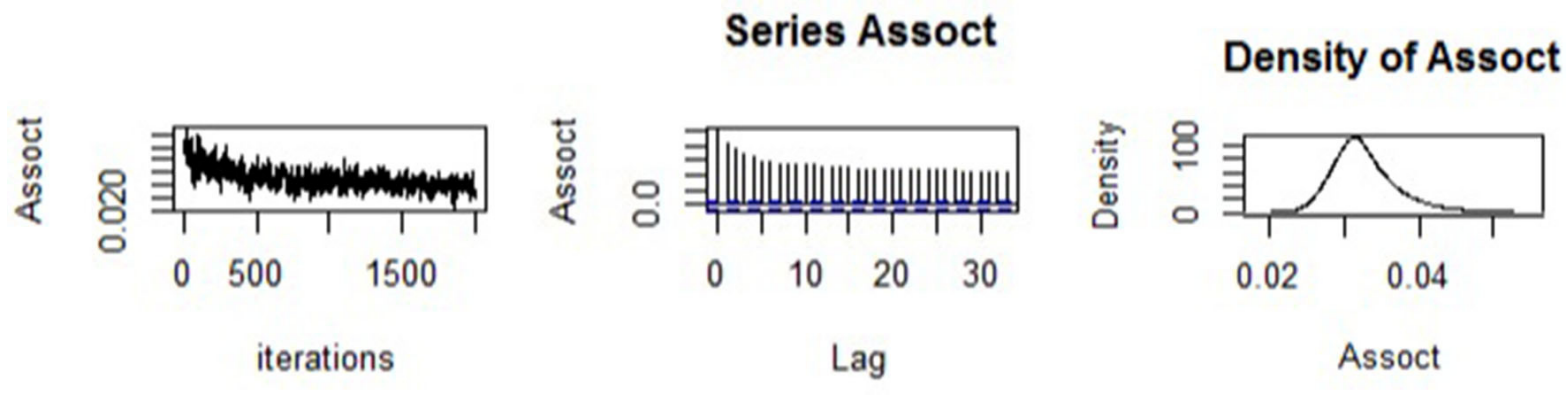
Trace, density, and autocorrelation diagnostic plots for the association parameter.

We begun by assessing the estimation quality of the fitted joint model. The trace plot, autocorrelation plot, and density plot for the association parameter are shown in Figure 1. The trace plot showed random plot around the mean value suggesting that the chains mixed well, which meant that parameter estimates across various iterations were within range. The autocorrelation plot was observed to become small and moving closer to zero (i.e., with small lags), meaning that the solutions of the simulated samples became quickly independent. Lastly, the density plot was observed to be unimodal and contain small tail. This provided confidence in the results of the estimated model parameters obtained.

### 3.2. Model Estimates

The results for the joint model are presented in Table 2. We report the posterior means of the estimated parameters, with their 95% credible intervals. We observed that the association between the longitudinal process and the survival outcome, α is 1.033 (CI: [1.026 - 1.044]) which is positive and significantly different from zero an indication that sputum smear result pattern is important in explaining the risk of unfavorable outcome. Specifically, this was interpreted as follows: The estimate of the association parameter α = 1.033 denoted the log hazard ratio for a unit increase in the log odds of having smear positive results. Sex, age, HIV status, BMI category, and TB treatment history were all found to be statistically significant in explaining the risk to unfavorable outcome among TB patients. Female patients reduced the risk to unfavorable outcomes by 0.81 (CI: [0.78–0.84]) compared to male. A unit increase in age increased the risk to unfavorable outcomes by 1.012 (CI: [1.011–1.014]). Compared to all other categories of BMI classification, patients with severe malnutrition were shown to have higher risk of unfavorable outcomes. Being HIV negative reduced the risk to unfavorable outcome by 0.47 (CI: [0.46–0.49]) compared to HIV-positive patients. Patients who have had previous episode of TB were 2.52 (CI: [2.41–2.63]) times more likely to experience unfavorable outcome.

**Table 2:** Model estimates.

**Covariate**		**Mean**	**Standard deviation**	**95% credible interval**
Age		1.013	0.0006	1.012–1.014^*^
Sex	Female	0.818	0.018	0.780–0.839^*^
HIV status	Negative	0.472	0.017	0.456–0.488^*^
Treatment history	Previously treated	2.520	0.022	2.412–2.630^*^
BMI category	MM	0.708	0.022	0.679–0.738^*^
	Normal	0.579	0.022	0.556–0.605^*^
	Overweight	0.511	0.050	0.462–0.562^*^
	Obese	0.432	0.004	0.382–0.482^*^
Association parameter		**1.033**	0.004	1.026–1.044^*^

* *Statistically significant at 0.05*.

### 3.3. Dynamic Predictions

**Figure 2:**
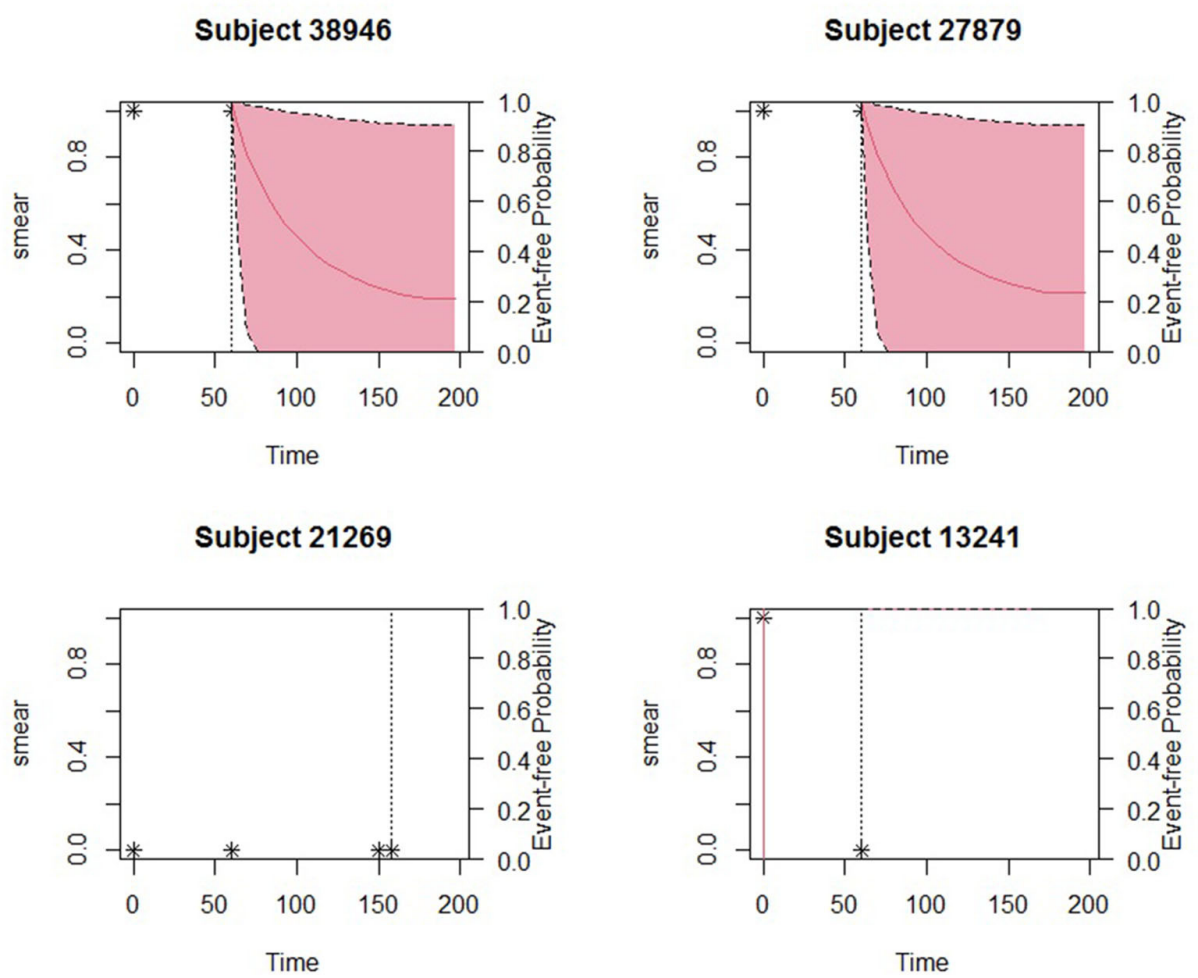
Predicted survival probabilities for patients 27879, 38946, 21269, and 13241. Solid red line is the mean of 2,000 MCMC samples. Dashed lines are the 2.5 and 97.5% percentiles range of the 2,000 MCMC samples. The dotted vertical line represents the time of prediction *t*.

**Figure 3:**
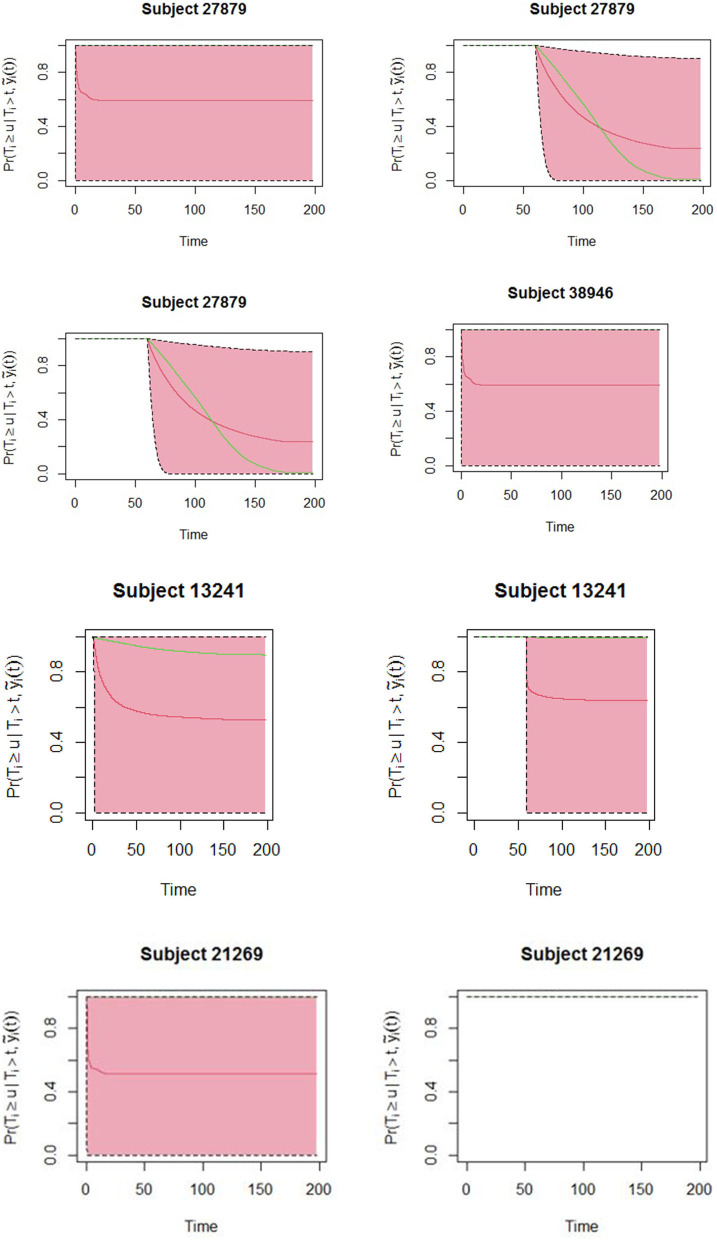
Dynamic predictions for patients 27879, 38946, 21269, and 13241. Solid red line is the mean of 2,000 MCMC samples. The green line is the true observed survival probabilities. Dashed lines are the 2.5 and 97.5% percentiles range of the 2,000 MCMC samples.

The obtained joint model was then used to predict subject-specific survival probabilities for 4 patients who had been randomly sampled from the study data and excluded in model building. Patient number 27879 was male, newly diagnosed, HIV status was negative, severely malnourished, and 19 years old. Patient number 38946 was female newly diagnosed, co-infected with HIV, severely malnourished, and 30 years old. Patient number 13241 was female newly diagnosed, HIV status was positive, severely malnourished, and 21 years old. The fourth patient was female newly diagnosed, HIV status negative, normal BMI, and 22 years old. Figure 2 displays the predicted survival probabilities based on different amounts of data. We observe that patients 27879 and 38946 both with month 0 and month 2 smear result as positive showed very low survival probabilities. Patient 21269 with consistent sputum smear negative results showed good survival probabilities. Patient 13241 had smear conversion after 2 months of treatment was shown to have good survival probabilities. Figure 3 displays the dynamic predictions as and when additional information is provided. The red line gives the predicted survival probabilities while the green line give the true observed survival probability. When only the baseline information is used for prediction, we observe that all the four patients have almost similar survival probabilities (near 50%). In addition, the predicted subject-specific probabilities are biased with wide uncertainty bands. By providing additional follow up sputum, we observe the following: The prediction for patient 27879 shifts down and closer to the true observed outcome. Similarly, the predictions for patient 38946 shifts down and closer to the true observed outcome. This indicates a higher risk of unfavorable outcomes for patients who do not smear convert by end of second month of treatment. For patient 13241, with baseline characteristics, we observe that our model under-predicted the survival probabilities. With additional second-month sputum smear results, the predictions slightly improve closer to the true observed outcome. The predicted values for patient 21269 were inline with the observed true value demonstrating very good survival probabilities.

## 4. Discussions

The aim of the study was to apply joint modeling approach to the repeated measures and survival outcomes from TB data. The advantage of this approach is on the inclusion of sputum smear results into the survival model. We have demonstrated the usefulness of sputum smear results in obtaining the risk of experiencing unfavorable outcome and in dynamically predicting individual survival through the treatment duration. The study considered sex, age, HIV status, BMI, and treatment history as baseline covariates and became part of a first survival analysis. In another part, a longitudinal analysis, where the sputum smear markers observed within 6 months of treatment for each subject characterized smear pattern until event occurrence. Both models were considered jointly, thus establishing an association parameter between the longitudinal and the survival processes. The use of joint modeling techniques was key in establishing whether sputum smear results also implied a simultaneous increased risk to unfavorable outcome for the subject. While being female was seen to be protective of unfavorable outcome, this is most likely explained by the poor health seeking behaviors among male populations (37, 38). In addition, possible explanations could be delayed treatment initiation among the males (8) and possibly non-adherence to treatment. The study did find HIV status to be important predictor to experiencing unfavorable outcome among TB patients. The findings were consistent with findings from (6, 39). Also, there was a significant association between treatment history and treatment outcome, an inconsistent finding with (40). In clinical practice, patients and clinicians will always be interested to know the survival probabilities during TB treatment. Dynamic prediction showed that different patients depending on their baseline characteristics and smear pattern across the treatment curve would have different survival probabilities. These predictions tend to be more accurate when additional sputum smear results are provided. Such predictions are very important to guide clinicians in tracking the health conditions of their patients and making individually informed decisions. Patient 13241 who had sputum smear conversion after 2 months have been shown to have very good survival probabilities beyond treatment duration, while patients 27879 and 38946 who do not convert are almost likely to experience unfavorable outcomes that include death or lost to follow up. Clinicians may therefore consider setting up control measures that will avert patients from these adverse outcomes. Although the results showed important relationship between the repeated measures of sputum smear trajectories and survival probabilities among TB patients, these findings are based on secondary data from routine surveillance hence possible inaccuracies. The study included only patients who had an initial smear result of either positive or negative. Lastly, additional repeated measures such as weight (collected at facility level but not relayed to national level) could improve the model predictions.

We believe that these findings will be of much interest to health care workers managing TB patients as well as National TB Program to guide in policy decisions.

## 5. Conclusion

The study concluded that, we conclude that sputum smear results is important in explaining the risk of unfavorable outcome and hence the need, going forward to analyze the time to event and endogenous processes simultaneously. Also TB patients who are male, co-infected with HIV, severely malnourished and have had previous history of TB are at higher risk of unfavorable outcomes. Different sputum smear pattern through the treatment curve demonstrated dynamic survival probabilities. Therefore, for patients with high predicted risks, clinicians may consider differentiated approaches such as closely monitoring adherence and provision of nutritional support to TB patients so as to avert unfavorable outcomes.

## Data Availability Statement

The data analyzed in this study is subject to the following licenses/restrictions: The data analyzed in this study is subject to authorized access only. Requests to access these datasets should be directed to Dr. Elizabeth Onyango/Division of National Tuberculosis, Leprosy and Lung Disease, eonyango@nltp.co.ke.

## Ethics Statement

Ethical review and approval was not required for the study on human participants in accordance with the local legislation and institutional requirements. Written informed consent from the participants' legal guardian/next of kin was not required to participate in this study in accordance with the national legislation and the institutional requirements.

## Author Contributions

RK, MK, AM, JK, and MO: conceptual and design of study and analysis and interpretation of data. EO and RK: acquisition of data. RK, MK, AM, and JK: drafting the manuscript. RK, MK, AM, JK, MO, and EO: revising the manuscript critically for important intellectual content and approval of the version of the manuscript to be published. All authors contributed to the article and approved the submitted version.

## Conflict of Interest

The authors declare that the research was conducted in the absence of any commercial or financial relationships that could be construed as a potential conflict of interest.
